# The prevalence and associated factors of dysphagia in Parkinson's disease: A systematic review and meta-analysis

**DOI:** 10.3389/fneur.2022.1000527

**Published:** 2022-10-06

**Authors:** Siyuan Gong, Yan Gao, Jihong Liu, Jia Li, Xueqin Tang, Qian Ran, Rongzhu Tang, Chunlian Liao

**Affiliations:** ^1^Department of Neurology, The Second Affiliated Hospital of Chongqing Medical University, Chongqing, China; ^2^Nursing Department, The Second Affiliated Hospital of Chongqing Medical University, Chongqing, China; ^3^Endocrinology Department, The Second Affiliated Hospital of Chongqing Medical University, Chongqing, China

**Keywords:** Parkinson's disease, dysphagia, prevalence, associated factors, meta-analysis

## Abstract

**Background:**

The prevalence and associated factors of dysphagia in Parkinson's disease (PD) are different in studies conducted in different countries. The purpose of our systematic review and meta-analysis was to evaluate the prevalence of dysphagia in PD and to clarify its associated factors.

**Methods:**

Two researchers systematically searched PubMed, Embase, Web of Science, Cochrane Library, CNKI, Wanfang Database, SinoMed and VIP databases and manually searched references in the retrieved articles to identify potential research subjects. The last search was conducted on June 28, 2022. Finally, a total of 58 studies including 60 observations with 20,530 PD patients were included in our meta-analysis.

**Results:**

The meta-analysis estimated that the pooled prevalence rate of dysphagia in PD was 36.9% (95% CI: 30.7–43.6%) and instrumental examination showed a higher prevalence (57.3%, 95% CI: 44.3–69.1%). Oceania showed the highest prevalence of dysphagia in PD (56.3%) compared to Africa (39.5%), Asia (38.6%), Europe (36.1%) and America (28.9%). Dysphagia in PD was associated with older age, lower body mass index, longer disease duration, higher Hoehn and Yahr stage and levodopa equivalent daily dose, PIGD subtype, severe motor symptoms, drooling and higher levels of depression, and lower quality of life.

**Conclusions:**

In conclusion, our meta-analysis showed that dysphagia occurs in more than one-third of PD patients and was associated with several demographic characteristics and PD-related characteristics, motor symptoms, non-motor symptoms, as well as decreased quality of life. It deserves early screening, diagnosis, and treatment in clinical practice to prevent serious complications from dysphagia.

## Introduction

Parkinson's disease (PD) is a neurodegenerative disease characterized by progressive degeneration of dopaminergic neurons in the substantia nigra of the midbrain and multiple system involvement (1). Dysphagia is a common non-motor symptom in patients with Parkinson's disease, which can lead to a range of serious problems such as malnutrition, aspiration pneumonia, prolonged hospital stay, and even increased mortality (2–4). Studies in different countries have reported large differences in the prevalence of dysphagia in PD, and prevalence of 32.0% in Israe (5) and 87.1% in China (6) have been reported. In previous studies, dysphagia in PD patients has been assessed by different methods such as standardized questionnaires (7), water swallowing test (8), interview (9), clinical assessment (10), and instrumental examination (11) etc., with a wide range of results. The review conducted by Kalf et al. reported the prevalence of dysphagia (12), but it was conducted 10 years ago, and many relevant studies have emerged in recent years. Representative estimates of the actual prevalence of dysphagia in PD are still scarce. Therefore, the present study is a necessary step in identifying and reporting on the prevalence of dysphagia in PD in the existing literature. In addition, previous studies have shown that its high prevalence is related to many factors, such as age, male sex, levodopa equivalent daily dose (LEDD), Hoehn and Yahr stage (H-Y stage) and cognitive impairment, etc. (6, 13, 14). However, the results of the association between dysphagia in PD and associated factors have been inconsistent across studies, and there have been few quantitative reviews to clarify the strength of these association. Understanding these related factors can enable clinicians and nurses to detect dysphagia in PD patients as soon as possible, and timely prevent and treat them, thereby preventing the occurrence of related complications. Therefore, a meta-analysis is needed to clarify the associated factors for dysphagia in PD. Given these gaps in the current research on dysphagia in PD, we conducted two systematic reviews with the following objectives: (1) to provide a comprehensive estimate of the prevalence of dysphagia in PD and (2) to calculate a summary effect estimate of the associated factors for dysphagia in PD by summarizing all available data. The results of this study can provide reference for clinical practice related to dysphagia in PD patients.

## Methods

### Study design and registration

Our meta-analysis adhered to the preferred reporting items for systematic reviews and meta-analyses (PRISMA) (15) statements and were registered with PROSPERO (registration number CRD42022344132).

### Search strategy

We searched PubMed, Embase, Web of Science, Cochrane Library, CNKI, Wanfang Database, SinoMed and VIP databases. Our search strategy was as follows: (Parkinson^*^ OR PD OR Paralysis Agitans) AND (dysphagia OR deglutition disorder^*^ OR swallowing dysfunction OR swallowing disorder OR impaired swallowing OR acataposis OR swallow problem^*^). The last search was conducted on June 28, 2022. In addition, we manually searched the references in the retrieved articles to identify potential research subjects. The detailed search strategy is illustrated in Supplementary Table 1.

### Selection criteria

A study was included if (1) patients were diagnosed with PD according to the United Kingdom Brain Bank Criteria (16), the Movement Disorders Society diagnostic criteria (17), or clinically diagnosed by neurologist; (2) dysphagia was assessed according to validated diagnostic or screening modality; (3) study reported the prevalence or associated factors of PD-related dysphagia or provided data that could estimate the prevalence or associated factors of it; (4) search design: cohort, case-control studies or cross-sectional; and (5) published in English or Chinese. Studies were excluded if they (1) were case reports, conference abstracts and reviews; (2) published in different studies with duplicate participants. If multiple studies reported results from the same sample including varying numbers of patients, we would consider the study with the larger sample size.

### Data extraction and quality assessment

Two authors (SY Gong and Y Gao) performed the literature screening alone according to the inclusion and exclusion criteria, respectively: (1) duplicate studies were removed using EndNote X9 software; (2) the titles and abstracts were screened to exclude obviously irrelevant literature; (3) the full text was read, and the reasons for exclusion were indicated; (4) the references were screened for inclusion. Then, two authors (SY Gong and Y Gao) independently extracted the data from each study in accordance with the predesigned data extraction. The quality of the included studies was assessed using the Newcastle Ottawa Scale (NOS) for cohort or case-control studies and the Agency for Healthcare Research and Quality (AHRQ) for cross-sectional studies. Based on the NOS score, the included eligible studies with 0–3, 4–6, and 7–9 points indicated low, moderate and high quality, respectively. Based on the AHRQ score, the included eligible studies with 0–3, 4–7, and 8–11 points indicated low, moderate and high quality, respectively. Disagreements were resolved through discussion and consultation with a third author (CL Liao). The extracted data included the characteristics of the following categories: author, publication year, country, age, disease duration, H-Y stage, evaluation tools, evaluation methods, dysphagia cases, sample size, dysphagia rate and associated factors.

### Statistical analysis

We used the software R software package version 4.1.1 to analyze all statistical data. In our study, in the meta-analyses focusing on the prevalence of dysphagia in PD, the effect sizes were defined as the prevalence of dysphagia in PD, If more than one method of assessing dysphagia was used in the same study, the results of the instrumental examination were used. In the meta-analyses of factors associated with dysphagia in PD, when more than 2 studies reported the same associated factor, the effect sizes were defined as the odds ratio (OR), the weighted mean difference (WMD) or standardized mean difference (SMD) and 95% CI. To account for the variability and heterogeneity of the prevalence among the included studies, we analyzed the data using a logit transformation random effects model (18). Due to a wide range of characteristics of the studies included, all analyses were performed using the random-effects model. Statistical heterogeneity between the studies was assessed using the *I*^2^ statistic, with *I*^2^ values of 25, 50, and 75% indicating low, moderate, and high heterogeneity, respectively (19). Moreover, in the meta-analyses focusing on the prevalence of dysphagia in PD, subgroup analyses were conducted on the continent and evaluation method. To estimate the stability of the overall results and distinguish the potential impact of individual studies, sensitivity analyses were performed by removing each study individually. In addition, the funnel plots and Egger's test were used to assess possible publication bias (20). A *P* < 0.05 was used for the comparison of the result variables, and the difference was statistically significant.

## Results

### Search results

**Figure 1:**
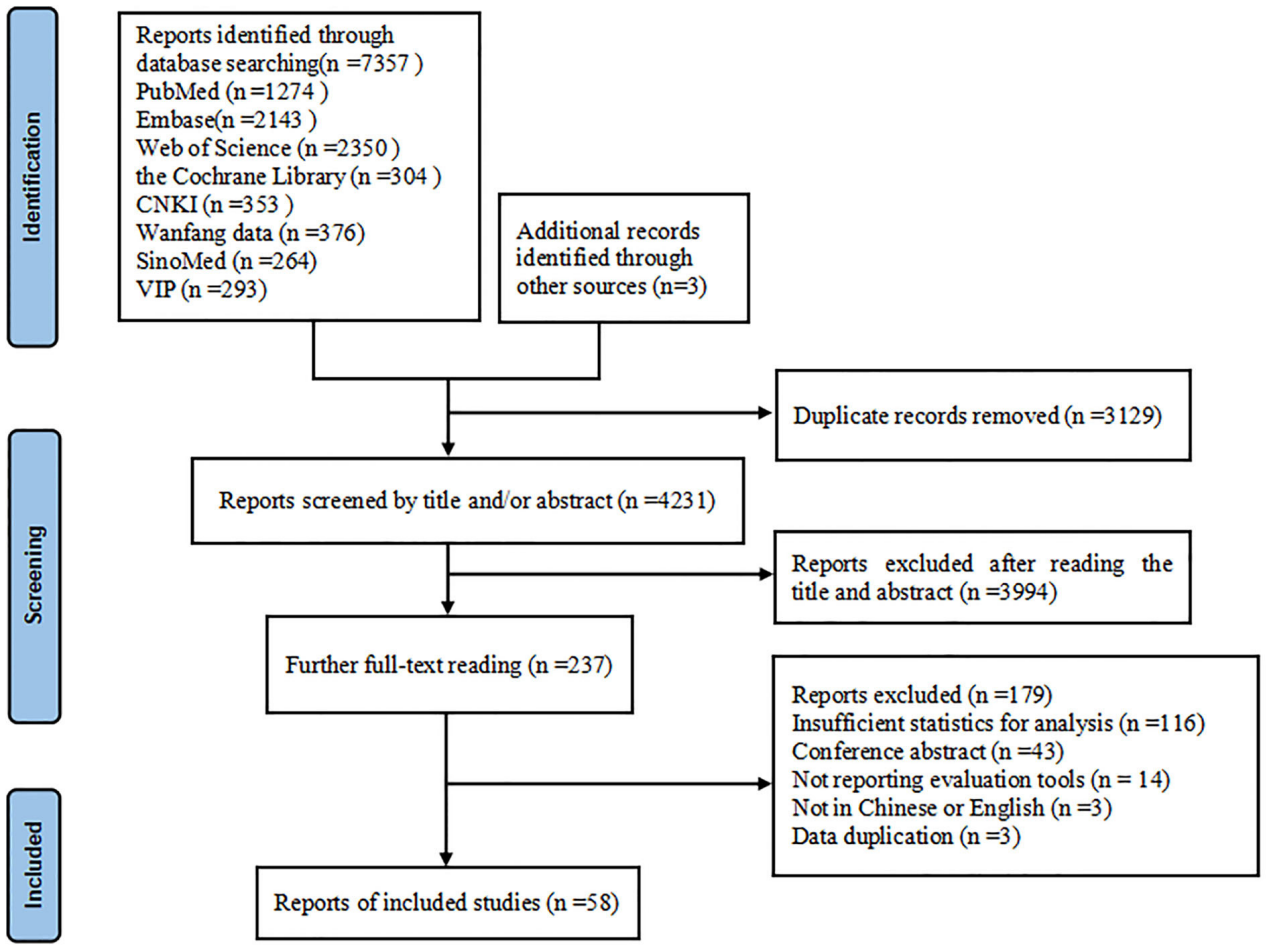
The specific inclusion screening process.

Our research initially retrieved 7,360 articles, of which 3,129 articles were removed due to duplication, and 4,231 articles remained. Among them, 3,994 articles were excluded because their abstracts and titles failed to meet the selection criteria; thus, 237 remained. After we read the full text of the articles, 179 articles were excluded for the following reasons: insufficient statistics for analysis, conference abstract, not reporting evaluation tools, not in Chinese or English, data duplication. Finally, 58 studies (5–11, 13, 14, 21–69) remained and were included in this research. The specific inclusion screening process is shown in Figure 1.

### Study characteristics

A total of 58 studies (5–11, 13, 14, 21–69) including 60 observations with 20,530 PD patients were included in the present study. Thirty-seven were cross-sectional studies, 20 were cohort studies, and 1 case-control study. These studies were published between 1997 and 2022, and their sample sizes ranged from 24 to 6,462. Twenty-seven studies were carried out in Asia, 23 in Europe, 5 in America, 2 in Africa and 1 in Oceania. All the included studies reported the prevalence of dysphagia in PD, 27 of which reported on associated factors. In total, 17 studies used instrumental examination as evaluation method, 8 studies used standardized questionnaires or scales, 3 studies used water swallowing test, and 30 studies used simple rating scores or interview, and dysphagia rate ranged from 7.2 to 95.0%. The quality assessment scores of the included studies ranged from 5 to 8 points (median 6 points) based on NOS, indicating moderate quality; and 2–8 points (median 6 points) based on AHRQ, also indicating moderate quality. The detailed NOS or AHRQ scores of the included studies are shown in Supplementary Tables 2, 3. The detailed characteristics of the included reports are described in Table 1.

**Table 1:** Detail characteristics of the included studies.

**Author**	**Country**	**Age**	**Disease duration (y)**	**H-Y stage**	**Evaluation tools**	**Evaluation methods**	**Dysphagia cases**	**Sample size**	**Dysphagia rate (%)**	**Associated factors**
Coates (21)	United Kingdom	69.9 ± 8.75	6.7 ± 5.75	NA	Chicago assessment scale	Standardized questionnaires or scales	43	48	89.6	NA
Clarke (22)	United Kingdom	66.7 ± 8.25	9 ± 10.5	3 ± 0.88	Speech therapist's global rating scale and Chicago rating scale	Standardized questionnaires or scales	19	64	29.7	NA
Siddiqui (23)	United States	65.6 ± 9.0	8.3 ± 6.5	2.1 ± 0.6	Gastrointestinal questionnaire	Simple rating scores or interview	13	44	29.5	NA
Martinez-Martin (24)	Spain	67.66 ± 10.46	6.96 ± 5.3	NA	NMSQ	Simple rating scores or interview	149	525	28.4	NA
Cheon (25)	Korea	64.9 ± 8.6	6.4 ± 6.1	2.33 ± 1.13	NMSQ	Simple rating scores or interview	23	74	31.1	1
Barone (9)	Italy	67.4 ± 9.4	5.67 ± 4.68	2 ± 0.74	Semistructured interview	Simple rating scores or interview	173	1,072	16.1	NA
Lo (26)	United States	70.5 ± 7.4	NA	NA	UPDRS	Simple rating scores or interview	108	466	23.2	NA
Manor (10)	Israel	67.06 ± 11.72	8.0 ± 6.1	2.06 ± 1.05	Clinical swallowing evaluation combined with FEES	Instrumental examination	38	69	55.1	2, 3, 4, 6, 7, 8
Miller (8)	England	72.5 ± 7.12	6.5 ± 5.62	2.33 ± 0.75	150 ml water swallow test	Water swallowing test	31	137	22.6	1
Zheng (27)	China	62 ± 9.5	5.5 ± 3.2	2.18 ± 0.89	NMSQ	Simple rating scores or interview	54	131	41.2	1
Walker (5)	Israel	75 ± 9.68	4.8 ± 4.26	2.54 ± 0.99	UPDRS	Simple rating scores or interview	24	75	32.0	1, 2, 3, 4, 6, 7, 8, 13
Perez-Lloret (28)	France	69 ± 10	6 ± 5	2.0 ± 0.5	UPDRS	Simple rating scores or interview	77	419	18.4	1, 11
Yamamoto (29)	Japan	67.0 ± 9.2	NA	3.18 ± 1.00	VFSS	Instrumental examination	9	61	14.8	1
Ma (30)	China	61.16 ± 11.44	5.28 ± 4.86	NA	NMSS	Simple rating scores or interview	121	440	27.5	NA
Barichella (7)	Italy	67.8 ± 9.2	8.8 ± 6.2	2.30 ± 0.73	SDQ	Standardized questionnaires or scales	23	208	11.1	2, 3, 9, 10, 12
Cersosimo (31)	Argentina	64.69 ± 8.75	7.91 ± 5.82	2.21 ± 0.46	Gastrointestinal questionnaire	Simple rating scores or interview	26	129	20.2	NA
Guo (32)	China	61.8 ± 11.8	4.5 ± 4.2	2.5 ± 0.9	NMSS	Simple rating scores or interview	119	616	19.3	NA
Cereda (33)	Italy	60.67 ± 11.12	7.67 ± 5.93	2.25 ± 0.89	NMSQ and UPDRS	Simple rating scores or interview	754	6,462	11.7	1
Rajaei (34)	Iran	66 ± 9.7	NA	1.98	VFSS	Instrumental examination	10	59	16.9	1, 2
Simons (11)	Germany	70.47 ± 8.4	11.1 ± 6.27	3.31 ± 0.74	FEES	Instrumental examination	56	77	72.7	NA
Lee (35)	Korea	72 ± 6.76	14.4 ± 7.49	1.67 ± 0.54	SDQ	Standardized questionnaires or scales	12	29	41.4	1, 2, 3, 4, 5, 6, 8, 10, 12
Ou (36)	China	61.94 ± 10.67	4.82 ± 4.12	2.0 ± 1.0	UPDRS	Simple rating scores or interview	188	518	36.3	11
Zhang (37)	China	61.54 ± 10.98	4.76 ± 4.18	2.35 ± 0.74	Interviews by physician	Simple rating scores or interview	160	454	35.2	NA
Li (38)	China	61.5 ± 11.02	5.38 ± 5.97	2.21 ± 0.64	NMSQ	Simple rating scores or interview	46	108	42.6	NA
Barbe (39)	Germany	71 ± 8.7	9.5 ± 6.4	NA	MDS-UPDRS	Simple rating scores or interview	46	99	46.5	NA
Fereshtehnejad (40)	Canada	64.7 ± 9.9	6.6 ± 5.5	NA	UPDRS	Simple rating scores or interview	32	314	10.2	NA
Malek (41)	England	67.6 ± 9.3	1.3 ± 0.9	NA	SCOPA-AUT	Simple rating scores or interview	349	1,738	20.1	NA
Wang (42)	China	64.7 ± 8.6	8.95 ± 4.86	1.68 ± 0.67	UPDRS	Simple rating scores or interview	23	42	54.8	NA
Ding (6)	China	64.1 ± 9.2	NA	NA	VFSS	Instrumental examination	101	116	87.1	1, 2, 13
Mohamed (43)	Egypt	62.30 ± 5.64	4.7 ± 2.2	2.1 ± 0.6	FEES	Instrumental examination	22	54	40.7	1, 2, 3, 4, 6, 9, 10, 11, 14
Mukhtar (44)	Pakistan	57.61 ± 10.64	NA	NA	NMSQ	Simple rating scores or interview	15	102	14.7	1
Barbe (45)	Germany	69 ± 8	8 ± 5	NA	Questionnaire	Simple rating scores or interview	50	75	66.7	NA
Fukuoka (46)	Japan	70.4 ± 7.9	7.3 ± 5.5	3.0 ± 1.3	VFSS	Instrumental examination	9	24	37.5	1, 2, 3, 4, 10
Nienstedt (47)	Germany	68.9 ± 10.1	9.7 ± 7.1	2.66 ± 0.92	FEES	Instrumental examination	113	119	95.0	NA
Oad (48)	New Zealand	72 ± 7	9 ± 5	NA	EAT-10	Standardized questionnaires or scales	40	71	56.3	NA
Paul (49)	India	63 ± 10.5	NA	NA	SDQ	Standardized questionnaires or scales	22	75	29.3	NA
Polychronis (50)	United Kingdom	61.62 ± 9.92	0.54 ± 0.54	1.56 ± 0.5	MDS-UPDRS	Simple rating scores or interview	49	398	12.3	1, 2, 4, 6, 7
Umay (51)	Turkey	63.08 ± 10.29	10.23 ± 5.59	2.06 ± 0.14	FEES	Instrumental examination	61	120	50.8	1, 2, 3, 4, 9, 10
Wang (52)	China	67.72 ± 8.59	5.13 ± 3.56	3.01 ± 1.1	VFSS	Instrumental examination	63	83	75.9	1, 2, 3, 4, 7, 8, 9, 10, 13
Bakhtiyari (53)	Iran	59.40 ± 4.79	5.81 ± 3.68	2.35 ± 0.86	Northwestern dysphagia patient check sheet	Standardized questionnaires or scales	43	85	50.6	NA
Claus (13)	Germany	68.2 ± 9.6	7.6 ± 5	2.8 ± 0.8	FEES	Instrumental examination	120	200	60.0	1, 2, 3, 4, 5, 10, 14
Fagerberg (54)	Germany	62.44 ± 7.97	7.71 ± 5.73	2.15 ± 0.47	Water swallow test	Water swallowing test	10	41	24.4	NA
Marano (55)	Italy	61.75 ± 9.68	5.76 ± 7.54	1.56 ± 0.51	SCOPA-AUT or MDS-UPDRS	Simple rating scores or interview	44	422	10.4	1, 2, 3, 6, 10, 12
Rascol (56)	France	67.8 ± 9.9	6.1 ± 4.9	NA	UPDRS	Simple rating scores or interview	127	671	18.9	11
Schrag (57)	United Kingdom	76.1 ± 8.4	15.4 ± 7.7	4.24 ± 0.63	UPDRS	Simple rating scores or interview	432	689	62.7	NA
van-Wamelen (58)	United Kingdom	65.72 ± 10.87	5.63 ± 5.08	2.19 ± 0.89	NMSS	Simple rating scores or interview	166	727	22.8	11
Van-Wamelen (58)	United Kingdom	68.98 ± 10.77	8.90 ± 5.37	2.58 ± 0.90	NMSS	Simple rating scores or interview	216	727	29.7	11
Lin (59)	China	63.26 ± 3.5	NA	NA	Water swallow test	Water swallowing test	43	75	57.3	NA
Wang (60)	China	67.44 ± 9.4	NA	NA	VFSS	Instrumental examination	48	71	67.6	NA
Xu (61)	China	71.49 ± 9.98	NA	NA	VFSS	Instrumental examination	25	61	41.0	1, 2, 12
Ayele (62)	Ethiopia	62.9 ± 10.4	4 ± 3	2.2 ± 1.1	NMSQ	Simple rating scores or interview	48	123	39.0	NA
Cao (63)	China	65.77 ± 12.97	4.18 ± 5.13	2.07 ± 0.97	Questionnaire	Simple rating scores or interview	16	221	7.2	NA
Frank (64)	Germany	69.8 ± 9.8	10.3 ± 9.2	2.61 ± 0.83	FEES	Instrumental examination	37	51	72.5	NA
Wang(a) (14)	China	61.63 ± 8.43	5.23 ± 4.80	1–5	VFSS	Instrumental examination	13	48	27.1	1, 2, 3, 6, 7, 8, 13
Wang(b) (14)	China	65.79 ± 8.65	9.50 ± 4.93	1–5	VFSS	Instrumental examination	29	48	60.4	1, 2, 3, 6, 7, 8, 13
Dilmaghani (65)	United States	70.13 ± 12.21	NA	2.93 ± 1.14	VFSS	Instrumental examination	105	138	76.1	NA
Longardner (66)	Italy	69.3 ± 9.4	17.8 ± 6.1	NA	MDS-UPDRS	Simple rating scores or interview	13	50	26.0	NA
Vogel (67)	Germany	66.67 ± 9.89	7.33 ± 3.8	3 ± 1.52	FEES	Instrumental examination	26	57	45.6	NA
Shi (68)	China	63.9 ± 9.2	4.8 ± 3.9	1–5	SDQ	Standardized questionnaires or scales	45	107	42.1	1, 2, 3, 5, 6, 7, 8, 9, 10, 11
Wang (69)	China	64.5 ± 6.8	2.4 ± 2.7	1–5	NMSQ	Simple rating scores or interview	53	203	26.1	1

NA, not available; NMSQ, Non-Motor Symptoms Questionnaires; UPDRS, Unified Parkinson's Disease Rating Scale; MDS-UPDRS, Movement Disorder Society-sponsored revision of the Unified Parkinson's Disease Rating Scale; EAT-10, Eating Assessment Tool-10; SCOPA-AUT, Scale for Outcomes in Parkinson's disease for Autonomic Symptoms; 1, Geder; 2, Age; 3, Disease duration; 4, H-Y stage; 5, LEDD; 6, Cognitive function; 7, Anxiety; 8, Depression; 9, UPDRS-II or MDS-UPDRS-II; 10, UPDRS-III or MDS-UPDRS-III; 11, Drooling; 12, Body Mass Index (BMI); 13, Parkinson's disease questionnaire-39 (PDQ-39); 14, Postural instability/gait difficulty (PIGD) subtype.

### Prevalence of dysphagia in Parkinson's disease

**Figure 2:**
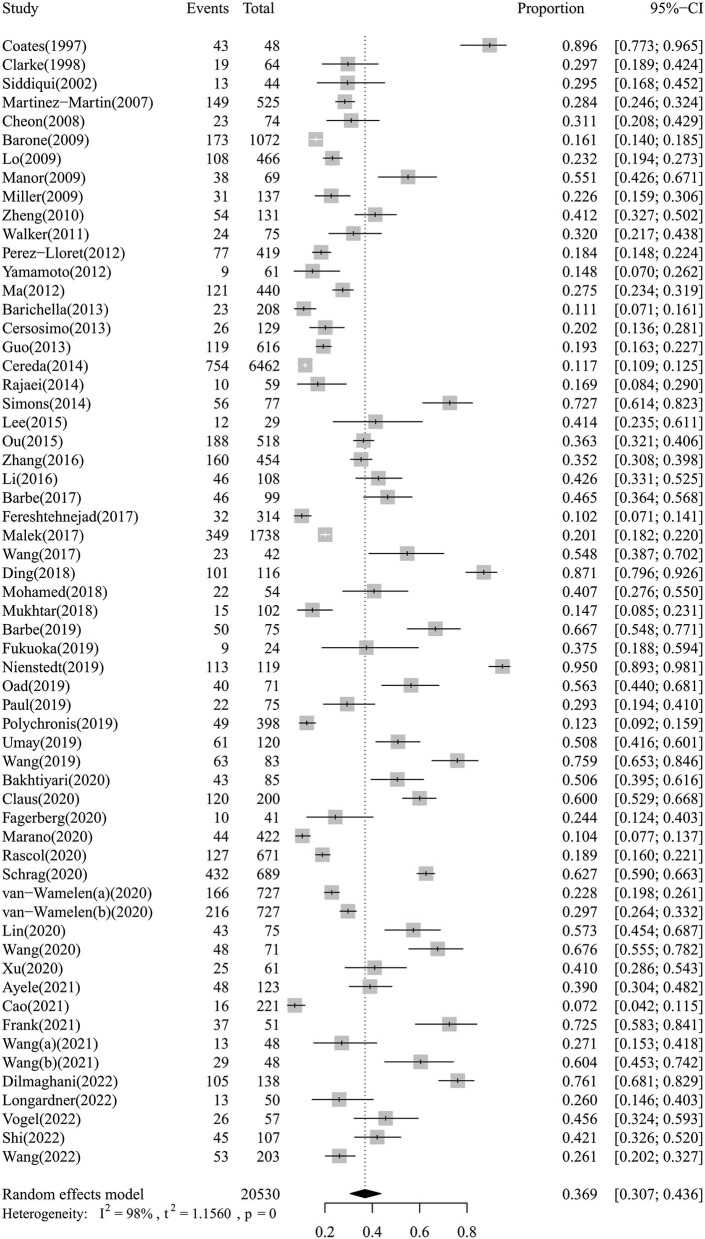
The forest plot of the overall prevalence of dysphagia in PD.

The meta-analysis of the overall prevalence of dysphagia in PD is shown in the forest plot in Figure 2. The random effects meta-analysis estimated that the pooled prevalence rate of dysphagia in PD was 36.9% (95% CI: 30.7–43.6%). There was high heterogeneity among the studies pooled for the meta-analysis (*I*^2^ = 98%). The *I*^2^ statistic describes the percentage of variation across the studies, and this is due to heterogeneity rather than a chance. The subgroup analyses were then performed by the continent and evaluation method. The subgroup analyses found that the evaluation method to be significant moderators, while the continent was not. Regarding the evaluation method (Supplementary Figure 1), the instrumental examination showed a higher prevalence of dysphagia in PD (57.3%, 95% CI: 44.3–69.1%) compared to the standardized questionnaires or scales (42.8%, 95% CI: 25.8–61.7%), the water swallowing test (33.6%, 95% CI: 18.2–53.3%), and the simple rating scores or interview (26.0%, 95% CI: 21.0–31.4%). Regarding the continent (Supplementary Figure 2), the Oceania showed a higher prevalence of dysphagia in PD (56.3%, 95% CI: 44.0–68.1%) compared to the Africa (39.5%, 95% CI: 32.6–46.9%), the Asia (38.6%, 95% CI: 30.8–46.9%), the Europe (36.1%, 95% CI: 25.0–48.8%), and the America (28.9%, 95% CI: 13.1–52.2%). The subgroup analyses of the prevalence of dysphagia in PD based on random-effect analysis are shown in Table 2.

**Table 2:** Subgroup analyses of the prevalence of dysphagia in PD based on random-effect analysis.

**Variable**	**No. of studies/ observations**	**Pooled estimate % (95% CI)**	***I*^2^ (%)**	***P*-value**
**Continent**				0.06
America	5/5	28.9 (13.1, 52.2)	98	
Europe	23/24	36.1(25.0, 48.8)	99	
Asia	27/28	38.6 (30.8, 46.9)	93	
Africa	2/2	39.5 (32.6, 46.9)	0	
Oceania	1/1	56.3 (44.0, 68.1)	-	
**Evaluation method**				**< 0.01**
Instrumental examination	17/18	57.3 (44.3, 69.1)	92	
Standardized questionnaires or scales	8/8	42.8 (25.8, 61.7)	93	
Water swallowing test	3/3	33.6 (18.2, 53.3)	92	
Simple rating scores or interview	30/31	26.0 (21.2, 31.4)	98	

CI, confidence interval.

**Figure 3:**
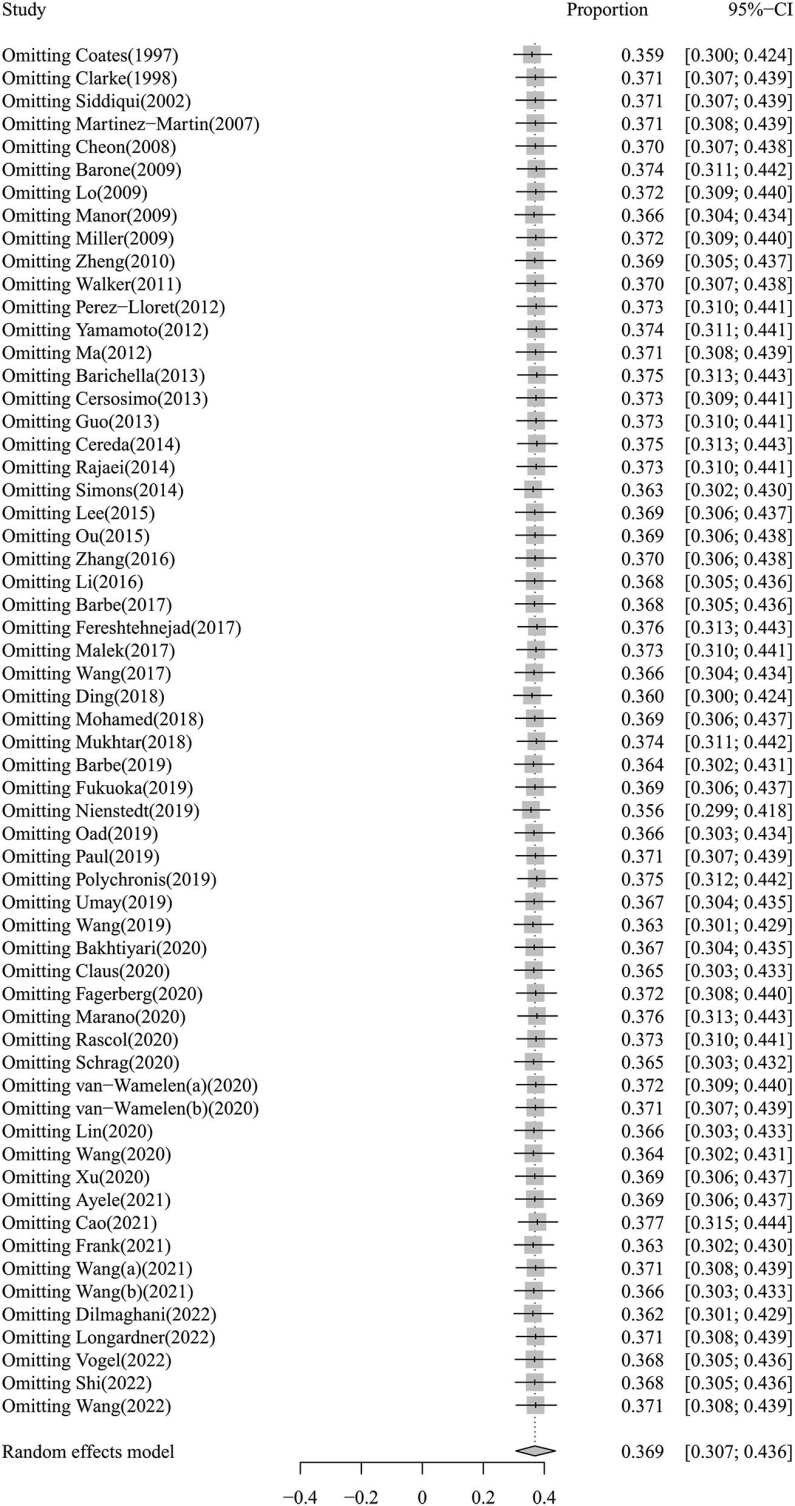
The forest plot for sensitivity analysis of the prevalence of dysphagia in PD.

The results of the sensitivity analysis indicated that our findings were robust and did not depend on a single study. Our pooled estimated prevalence of dysphagia in PD varied between 35.6% (95% CI: 29.9–41.8%) and 37.7% (95% CI: 31.5–44.4%) after removing each study individually. A forest plot for a sensitivity analysis of the prevalence of dysphagia in PD is shown in Figure 3. Regarding publication bias, visual inspection of the funnel plot indicated asymmetry (Supplementary Figure 3), and Egger's test also showed publication bias (*P* < 0.01).

### Factors associated with dysphagia in Parkinson's disease

In total, 27 studies (5–8, 10, 13, 14, 25, 27–29, 33–36, 43, 44, 46, 50–52, 55, 56, 58, 61, 68, 69) reported information about the associated factors in PD. Fourteen associated factors had data that could be used in the quantitative meta-analysis. Associated factors for included studies were summarized into the following main categories: demographic characteristics, such as gender, age, and BMI; PD-related characteristics, such as disease duration, H-Y stage, LEDD, and PIGD subtype; motor symptoms, such as UPDRS-II/MDS-UPDRS-II scores, and UPDRS-III/MDS-UPDRS-III scores; non-motor symptoms, such as cognitive function, anxiety, depression, and drooling; as well as quality of life. The meta-analyses of associated factors of dysphagia in Parkinson's disease based on random-effect analysis are shown in Table 3.

**Table 3:** Meta-analyses of associated factors of dysphagia in PD based on random-effect analysis.

**Factors**	**No. of studies/observations**	**Pooled MD/SMD/OR with 95%CI**	***I*^2^ (%)**	***P*-value for heterogeneity**
Gender (Male Yes vs. No)	22/23	1.13 (0.89–1.44)	52	< 0.01
Age (Mean ± SD)	16/17	2.97 (1.25–4.70)	67	< 0.01
BMI (Mean ± SD)	4/4	−1.70 (−3.21, −0.19)	63	0.04
Disease duration (Mean ± SD)	11/12	1.06 (0.56–1.56)	0	0.50
H-Y stage (Mean ± SD)	9/9	0.40 (0.17–0.63)	89	< 0.01
LEDD (Mean ± SD)	3/3	121.89 (38.81–204.98)	23	0.27
PIGD subtype (Yes vs. No)	2/2	3.09 (1.27–7.52)	0	0.42
UPDRS-II/MDS-UPDRS-II scores (Mean ± SD)	6/6	1.00 (0.60–1.41)	83	< 0.01
UPDRS-III/MDS-UPDRS-III scores (Mean ± SD)	10/10	0.40 (0.16–0.63)	66	< 0.01
Depression (Mean ± SD)	6/7	0.30 (0.08–0.52)	27	0.22
Cognitive function (Mean ± SD)	9/10	−0.27 (−0.56–0.02)	70	< 0.01
Anxiety (Mean ± SD)	5/6	0.20 (−0.24–0.64)	76	< 0.01
Drooling (Yes vs. No)	6/7	2.92 (2.46–3.45)	30	0.20
PDQ-39 (Mean ± SD)	4/5	7.06 (4.76–9.36)	33	0.20

WMD, Weighted mean difference; SMD, standardized mean difference; OR, odds ratio; CI, confidence interval.

### Demographic characteristics

Gender, Age and BMI. Twenty-two studies (5, 6, 8, 13, 14, 25, 27–29, 33–35, 43, 44, 46, 50–52, 55, 61, 68, 69) reported information about gender. But, our analysis did not find a significant association between PD with dysphagia and male gender (pooled OR: 1.13, 95% CI: 0.89–1.44) (Supplementary Figure 4A). Sixteen studies (5, 7, 10, 13, 14, 34, 35, 43, 46, 50–52, 55, 61, 68) reported the age of PD patients with dysphagia and those without dysphagia, and the meta-analysis showed that PD patients with dysphagia were older than those without dysphagia (pooled MD: 2.97, 95% CI: 1.25–4.70) (Supplementary Figure 4B). Four studies (7, 35, 55, 61) reported information about BMI. Our meta-analysis revealed that PD patients with dysphagia were lower BMI than those without dysphagia (pooled MD: −1.70, 95% CI: −3.21,−0.19) (Supplementary Figure 4C).

### PD-related characteristics

Disease duration, H-Y stage, LEDD and PIGD subtype. Of note, our analysis showed that longer disease duration (5, 7, 10, 13, 14, 35, 43, 46, 51, 52, 68), higher H-Y stage (5, 10, 13, 35, 43, 46, 50–52) and higher LEDD (13, 35, 68) were all associated with dysphagia in PD patients. The pooled MDs were 1.06 (95% CI: 0.56–1.56), 0.40 (95% CI, 0.17–0.63) and 121.89 (95% CI: 38.81–204.98), respectively (Supplementary Figures 5A–C). Moreover, two studies (13, 43) reported information about PIGD subtype. Our analysis found a significant association between PD with dysphagia and PIGD subtype (pooled OR: 3.09, 95% CI: 1.27–7.52) (Supplementary Figure 5D).

### Motor symptoms

UPDRS-II/MDS-UPDRS-II and UPDRS-III/MDS-UPDRS-III Scores. Our study found that PD patients with dysphagia had higher UPDRS-II/MDS-UPDRS-II scores (7, 43, 50–52, 68) and UPDRS-III/MDS-UPDRS-III scores (7, 13, 35, 43, 46, 50–52, 55, 68) than those without dysphagia. The pooled SMDs were 1.00 (95% CI: 0.60–1.41) (Supplementary Figure 6A) and 0.40 (95% CI, 0.16–0.63) (Supplementary Figure 6B), respectively.

### Non-motor symptoms

Depression, Cognitive function, Anxiety and Drooling. Our meta-analysis found that PD patients with dysphagia had slightly higher scores on depression scales (5, 10, 14, 35, 52, 68) than those without dysphagia (pooled SMD: 0.30, 95%CI: 0.08–0.52) (Supplementary Figure 7A). But, our analysis did not find a significant association between PD with dysphagia and cognitive function scores (5, 6, 10, 14, 35, 43, 50, 55, 68) and anxiety scores (10, 14, 52, 68). The pooled SMDs were −0.27 (95% CI: −0.56–0.02) (Supplementary Figure 7B) and 0.20 (95% CI, −0.24–0.64) (Supplementary Figure 7C), respectively. Of note, our meta-analysis of six studies (28, 36, 43, 56, 58, 68) showed that drooling was significantly associated with dysphagia in PD (pooled OR: 2.92, 95% CI: 2.46–3.45) (Supplementary Figure 7D).

### Quality of life

Four studies (5, 6, 14, 52) reported PDQ-39 scores in patients with dysphagia and those without dysphagia, and the meta-analysis showed that PD patients with dysphagia were significantly higher (worse) PDQ-39 scores than those without dysphagia (pooled MD: 7.06, 95% CI: 4.76–9.36) (Supplementary Figure 8).

## Discussion

### Prevalence of dysphagia in Parkinson's disease

The mechanism of dysphagia in PD is unclear. Studies have shown that in the progression of PD, a variety of pathological changes occur in the neuromuscular associated with swallowing function, resulting in impaired central and peripheral swallowing regulation mechanisms, and eventually swallowing disorders (70, 71). PD can impair all phases of swallowing (72). Dysphagia in the oral phase of PD patients is characterized by difficulties in the initiation of swallow, oral residues, piecemeal swallow and premature falling of the food; the pharyngeal phase is characterized by regurgitation of food into the nasal cavity or upper pharynx, pharyngeal residue and penetration/aspiration, and the esophageal phase is characterized by reduced esophageal peristalsis (71). The severity of dysphagia was assessed by FEES, with mild dysphagia presenting only with premature spillage and/or residues, and severe dysphagia presenting with frequent penetration/aspiration events (72). Although dysphagia become apparent in the later stages of PD, they may have been present in the early stages but often go undetected (73).

Our study established that dysphagia in PD is common. We found that the global prevalence of dysphagia in PD was 36.9%, the Oceania was 56.3%, the Africa was 39.5%, the Asia was 38.6%, the Europe was 36.1%, and the America was 28.9%. The high prevalence of dysphagia in PD reminds us that it is very important to screen out high-risk groups of dysphagia early to promote early clinical diagnosis and treatment, thereby preventing serious complications caused by dysphagia. It should be noted that there are few relevant studies from the Oceania and Africa. Therefore, more studies in these regions are warranted to further clarify the prevalence of dysphagia in PD and its associated factors.

In the subgroup analysis of evaluation method, the prevalence of dysphagia in PD patients assessed by simple rating scores or interview, water swallowing test, standardized questionnaires or scales, and instrumental examination increased sequentially (26.0: 33.6: 42.8: 57.3%). As expected, studies using more rigorous assessments reported a higher prevalence of dysphagia in PD patients. Instrumental examinations (VFSS and FEES) provide accurate assessment of swallowing function and are considered the “gold standard” for the diagnosis of dysphagia. VFSS can provide detailed assessment and analysis of the different phases of swallowing (oral and pharyngeal and esophageal phase), as well as the anatomical structures of the tongue, soft palate, pharynx and larynx and the delivery process of the food mass, which is crucial in the diagnosis of silent aspiration (74, 75). FEES can detect residue, penetration and aspiration by observing the function of the structures of the nose, pharynx and larynx during food swallowing and the location and amount of pigmented food mass remaining during feeding (76–78). Our research revealed that only about half of dysphagia were detected by simple rating scores or interview (26.0%), compared with instrumental examination (57.3%). These simple rating scores or interviews rely on the PD patient's self-perception of swallowing and are not reliable modality for identifying dysphagia, consistent with the results of previous studies (47, 79). Therefore, the use of simple rating scores or interview to screen PD patients for dysphagia is not recommended in future studies. Our study showed that the prevalence of dysphagia detected using the water swallowing test was much lower than that of instrumental examination (33.6 vs. 57.3%). The diagnostic accuracy of water swallowing test relies on the retention of cough reflex and pharyngeal-laryngeal sensitivity (80). However, PD patients often have a weak cough reflex and silent aspiration, and the water swallowing test may underestimate the prevalence of dysphagia. The combination of water swallowing test with clinical tests to assess voluntary and/or reflex cough function may be considered in clinical applications to improve accuracy (81). In addition, our study found that the prevalence of dysphagia in PD patients assessed using standardized questionnaires or scales were second only to instrumental examination (42.8 vs. 57.3%). Standardized questionnaires or scales that combine multiple aspects of clinical signs and symptoms, social life, or psychological implications associated with dysphagia, may be considered valid tools for screening for dysphagia (80). Although instrumental examination is indeed an objective and crucial modality for the diagnosis of dysphagia, its clinical application is limited due to its relatively expensive and the limitations of instrumentation and technology (47, 82). If clinical conditions allow, instrumental examination is recommended as the first choice to assess swallowing function in PD patients; if clinical conditions are limited, especially in the epidemiological investigation or screening of dysphagia in PD patients, it may be simpler and more convenient to use standardized questionnaires or scales. If the screening result is positive, further clinical evaluation or instrumental examination should be carried out by specialists to confirm the presence and assess the severity of dysphagia in order to avoid the occurrence of serious complications.

### Factors associated with dysphagia in Parkinson's disease

Our study compared PD patients with and without dysphagia showed that PD patients with dysphagia were older and associated with lower BMI. With age, decreased muscle tone in the elderly can lead to decreased chewing and swallowing function, and more prone to dysphagia (83). Several previous studies have also shown that age is significantly associated with dysphagia (84, 85). BMI not only partially reflects the nutritional status of patients, but also correlates with dysphagia. A study by Sakamoto et al. showed that a smaller BMI was significantly associated with dysphagia (86). The review conducted by Simons also showed that low BMI is a highly correlated clinical predictor of severe dysphagia in PD patients (87). This may be related to reduced energy intake due to dysphagia, which in turn leads to weight loss (88). In the present meta-analysis, we observed no significant association between male gender and the prevalence of dysphagia in PD patients based on the random effects model, with moderate heterogeneity (*I*^2^ = 52%). However, previous review has shown that gender differences in pathophysiological characteristics of PD patients are manifested by males having greater impairment in the etiology of the disease, possibly due to increased physiologic striatal dopamine levels due to estrogenic activity, which decreases incidence and age of onset in females (89). A study by Dumican et al. showed significantly higher (worse) overall pharyngeal phase dysphagia severity Videofluoroscopic Dysphagia Scale (VDS) scores in men with PD compared to women (90). The inconsistency of our study with the results of previous studies may be related to the inclusion of different swallowing phases in this study, which showed moderate heterogeneity. The association between gender and the different phases of dysphagia in PD patients needs to be further explored in the future.

Our meta-analysis showed that PD patients with dysphagia had significantly longer disease duration, higher H-Y stage, and higher LEDD than patients without dysphagia. These PD-related characteristics factors all predict further progression of PD or more severe symptoms. The lack of dopamine in the striatum may impair the intramedullary swallowing network in PD patients. As the disease progresses in PD patients, dopaminergic decline continues, which may lead to a decline in the function of the swallowing system (2, 13). Dysphagia occurs in nearly all patients with advanced PD, and is closely related to the severity of PD (12, 91). Of note, our study revealed that PD patients with PIGD subtype were more vulnerable to dysphagia compared to other PD subtypes. Previous study has also indicated that dysphagia may be more generalized in the PIGD subtype (8), and these findings may be the result of poor coordination of the oropharyngeal muscles with rigidity and prolonged oral transit time (92).

In the present meta-analysis, we observed that PD patients with dysphagia had significantly more severe motor symptoms than without dysphagia. The UPDRS-II/MDS-UPDRS-II score includes the screening items for dysphagia, and this part of the score is closely related to the occurrence of dysphagia. In addition, the results of this study also suggested that PD patients with dysphagia had higher UPDRS-III/MDS-UPDRS-III scores, consistent with previous studies (87). Therefore, PD patients should be closely monitored for the occurrence of dysphagia when aggravation of motor symptoms is found clinically.

Our meta-analysis revealed that PD patients with dysphagia were significantly more depressed than patients without dysphagia. The study conducted by Han et al. suggested that depression levels became more severe as the frequency of dysphagia increased, and depression is a predictor of dysphagia, which may also be a precursor to depression (93). This may be related to the possibility of various oral and pharyngeal symptoms in PD patients with dysphagia, resulting in psychological fear and stigma. In the present meta-analysis cognitive function was not related to dysphagia in PD patients based on the random effects model, with high heterogeneity (*I*^2^ = 70%). However, previous studies have shown that dysphagia is associated with the classification of cognitive impairment (93). The study conducted by Yatabe et al. also showed that cognitive decline may be an independent predictor of dysphagia (94). The results of the present study were inconsistent with those of the afore-mentioned studies, which may be attributed to the inconsistency of the included study population (the afore-mentioned study included an elderly population), and the results of the cognitive function assessment in our study with the Montreal Cognitive Assessment (MoCA) and Mini-Mental State Examination (MMSE) scale scores, respectively, without exploring the classification of cognitive impairment.

Our analysis did not find a significant association between PD with dysphagia and anxiety based on the random effects model, with high heterogeneity (*I*^2^ = 76%). Previous studies have suggested that anxiety in PD should be assessed with tailored screening tools (95). But, the anxiety scores included in our study were all general scales. Tailored screening tools may be needed to further explore the correlation between dysphagia and anxiety in patients with PD. Our study revealed that PD patients with drooling were more vulnerable to dysphagia, compared with PD patients without drooling. Dysphagia in PD patients is associated with oropharyngeal muscle dyskinesia and muscle stiffness caused by damaged basal ganglia (96). The dyskinesia of the throat muscles may be the common pathogenesis of salivation and dysphagia (28). In addition, study has found that the cause of salivation in PD patients may be oral retention caused by related dysphagia, suggesting that salivation is at least a manifestation of subclinical dysphagia (97).

Last, our meta-analysis observed that PD patients with dysphagia had significantly lower quality of life than PD patients without dysphagia. The progression of dysphagia may lead to longer meal times and dietary restrictions, negatively impacting the quality of life of PD patients (80). Several studies have reported that dysphagia can have a negative impact on psychological and social integrations (98, 99), which may indirectly reduce the quality of life of PD patients. In addition, the exacerbation of motor and non-motor symptoms due to difficulty in taking oral antiparkinsonian therapy is another problem that often affects PD patients' quality of life (100). Previous studies have also found that the severity of dysphagia significantly affects the quality of life of PD patients (101, 102). Therefore, in clinical work, early diagnosis and treatment of dysphagia is important to improve the quality of life of PD patients.

## Strengths and limitations

To our knowledge, this was the first and most comprehensive meta-analysis to include both the prevalence and associated factors of dysphagia in PD. However, the current meta-analysis had several limitations. First, in a meta-analysis of the prevalence of dysphagia in PD, we observed high heterogeneity and publication bias. The heterogeneity across the studies and publication bias may result from the differences in the study settings, samples and diagnostic methods. Second, in the meta-analysis of the associated factors of dysphagia in PD, we included the associated factors that were not used and adjusted for them in the multivariate analysis, and we did not perform a subgroup analysis, which may lead to some potential bias. Third, our meta-analysis included only those studies published in English or Chinese and ignored studies in other languages. Fourth, we did not search the gray literature, and there may be potential studies that were not included. Fifth, limited by the inconsistent definition of dysphagia severity in the included studies, our study only discussed clinical factors related to PD patients with or without dysphagia, and clinical factors related to the severity and specific phase of dysphagia need to be further explored in the future.

## Conclusions

In conclusion, our meta-analysis showed that dysphagia occurs in more than one-third of PD patients and was associated with several demographic characteristics, PD-related characteristics, motor symptoms, non-motor symptoms as well as lower quality of life. It deserves early screening, diagnosis, and treatment in clinical practice to prevent serious complications from dysphagia in PD and improve the quality of life.

## Data availability statement

The original contributions presented in the study are included in the article/Supplementary material, further inquiries can be directed to the corresponding author/s.

## Author contributions

SG and CL contributed to the design. SG and YG helped in statistical analysis and participated in most of the study steps. JLiu, JLi, and XT prepared the manuscript. RT and QR assisted in designing the study and helped in the interpretation of the study. All authors have read and approved the content of the manuscript.

## Conflict of interest

The authors declare that the research was conducted in the absence of any commercial or financial relationships that could be construed as a potential conflict of interest.

## Publisher's note

All claims expressed in this article are solely those of the authors and do not necessarily represent those of their affiliated organizations, or those of the publisher, the editors and the reviewers. Any product that may be evaluated in this article, or claim that may be made by its manufacturer, is not guaranteed or endorsed by the publisher.

## Supplementary material

Supplementary Figure 1 The forest plots for subgroup analysis on the evaluation method of the prevalence of dysphagia in PD based on random-effect analysis.

Supplementary Figure 2 The forest plots for subgroup analysis on the continent of the prevalence of dysphagia in PD based on random-effect analysis.

Supplementary Figure 3 The funnel plot for included studies on prevalence of dysphagia in PD.

Supplementary Figure 4 The forest plot for gender **(A)**, age **(B)**, BMI **(C)** based on random-effect analysis.

Supplementary Figure 5 The forest plot for disease duration **(A)**, H-Y stage **(B)**, LEDD **(C)**, PIGD subtype **(D)** based on random-effect analysis.

Supplementary Figure 6 The forest plot for UPDRS-II/MDS-UPDRS-II scores **(A)**, UPDRS-III/MDS-UPDRS-III scores **(B)** based on random-effect analysis.

Supplementary Figure 7 The forest plot for depression **(A)**, cognitive function **(B)**, anxiety **(C)**, drooling **(D)** based on random-effect analysis.

Supplementary Figure 8 The forest plot for PDQ-39 scores based on random-effect analysis.

Supplementary Table 1 The detailed search strategy.

Supplementary Table 2 Quality assessment based on NOS.

Supplementary Table 3 Quality assessment based on AHRQ.
