# Supplementary figures and images for: The prevalence and associated factors of dysphagia in Parkinson's disease: A systematic review and meta-analysis
Source: Front Neurol. 2022 Oct 6;13:1000527. doi: 10.3389/fneur.2022.1000527 (PMC9582284; doi:10.3389/fneur.2022.1000527)

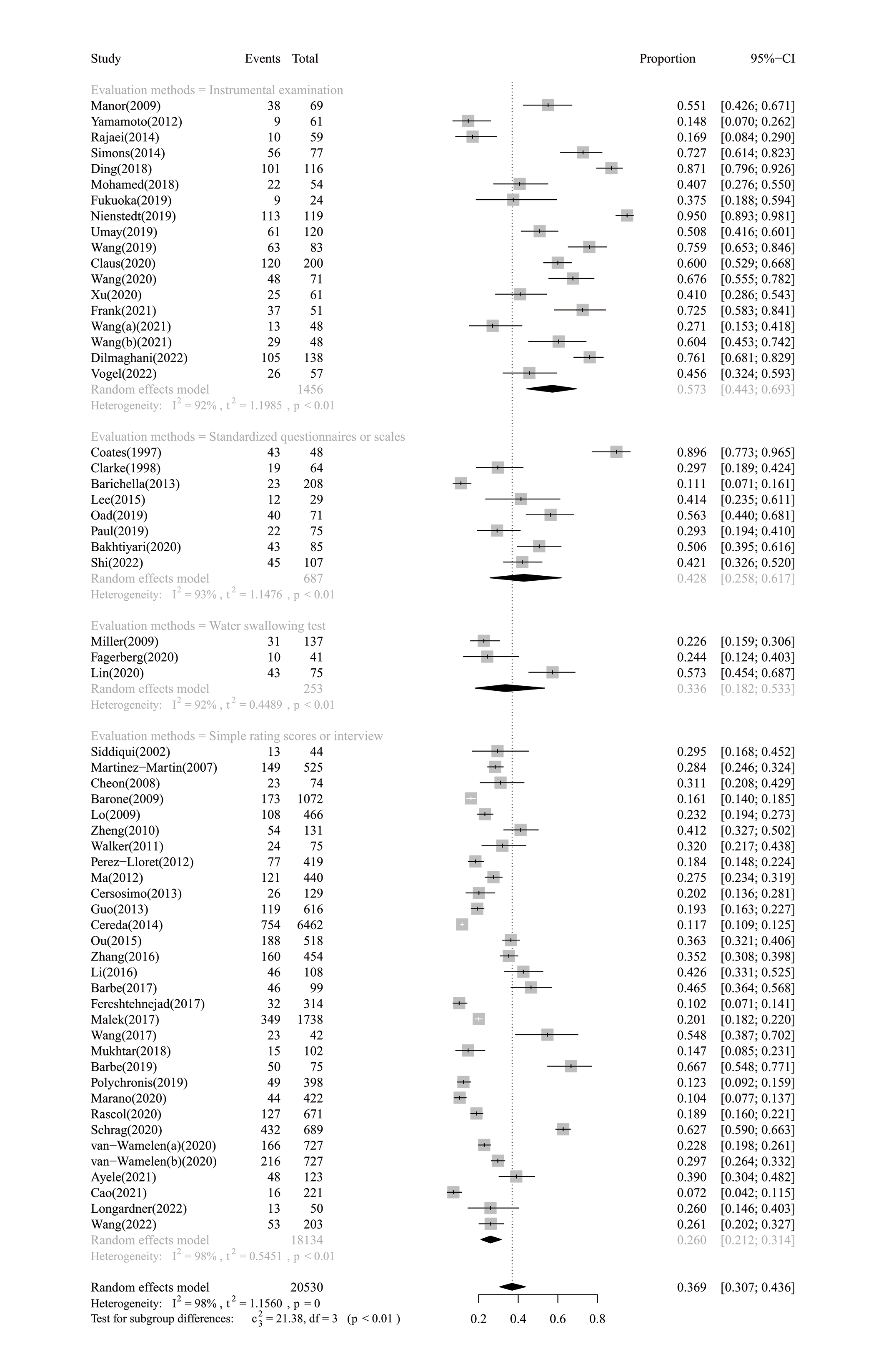

Supplement: Supplementary Figure 1 — The forest plots for subgroup analysis on the evaluation method of the prevalence of dysphagia in PD based on random-effect analysis. [file Image_1.TIF]

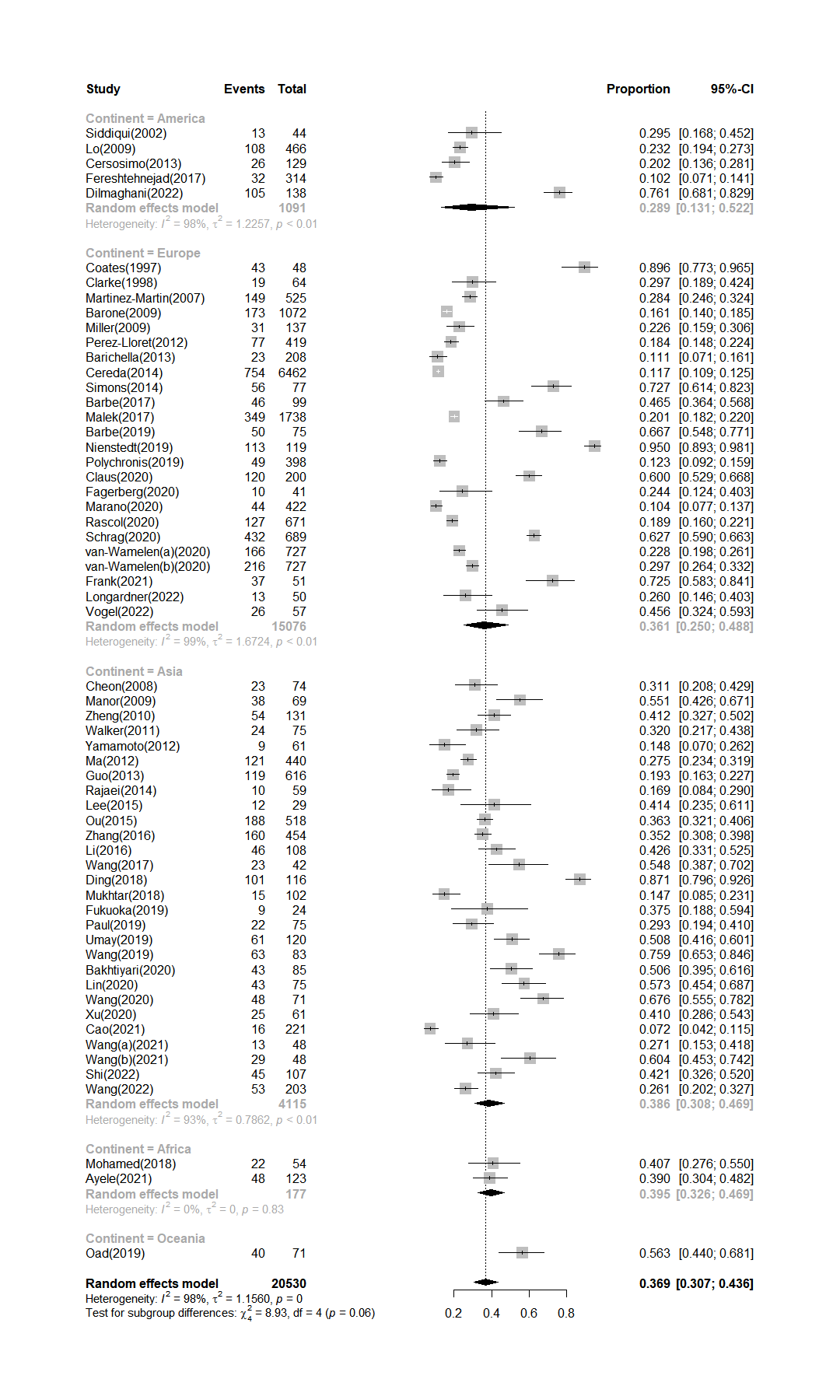

Supplement: Supplementary Figure 2 — The forest plots for subgroup analysis on the continent of the prevalence of dysphagia in PD based on random-effect analysis. [file Image_2.TIFF]

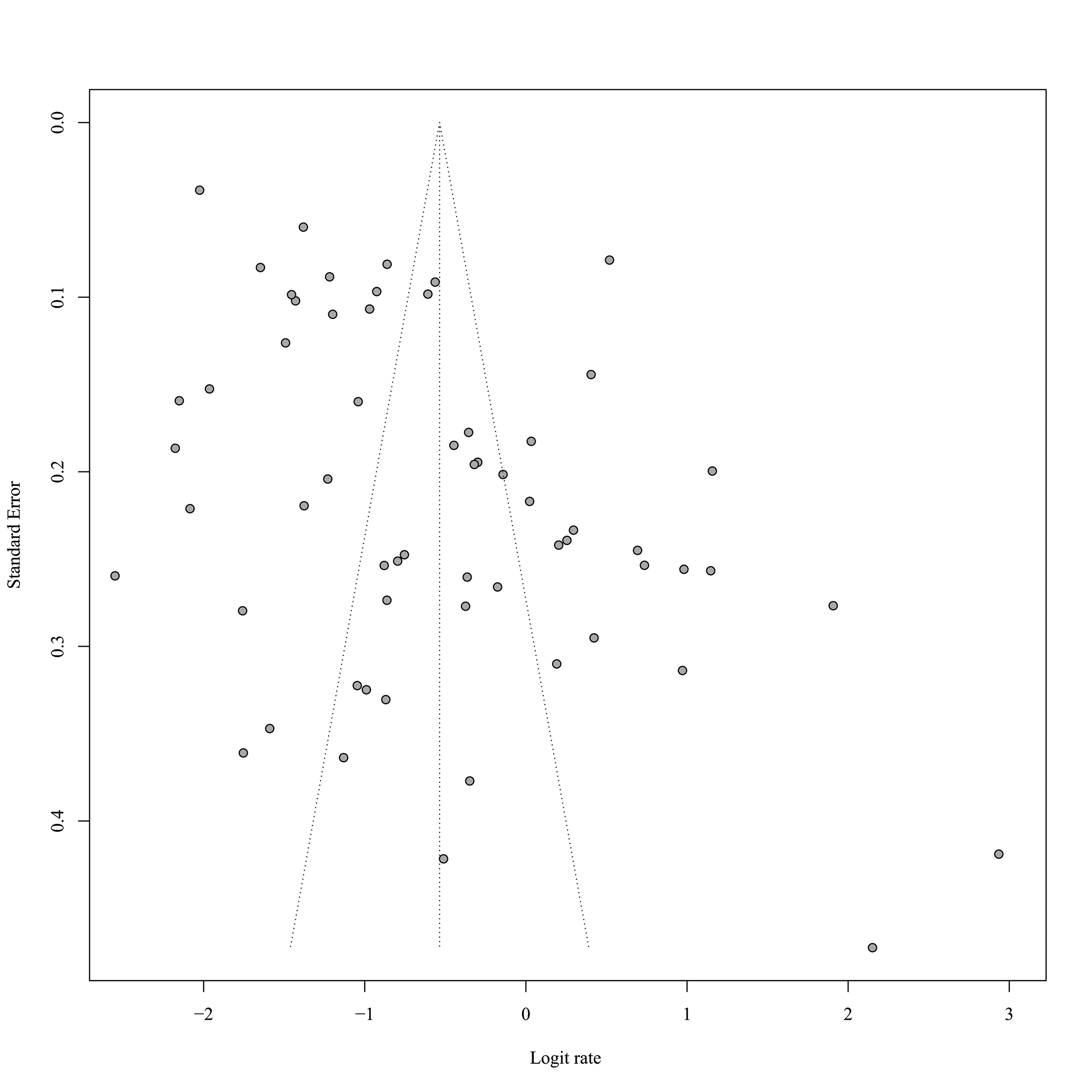

Supplement: Supplementary Figure 3 — The funnel plot for included studies on prevalence of dysphagia in PD. [file Image_3.TIF]

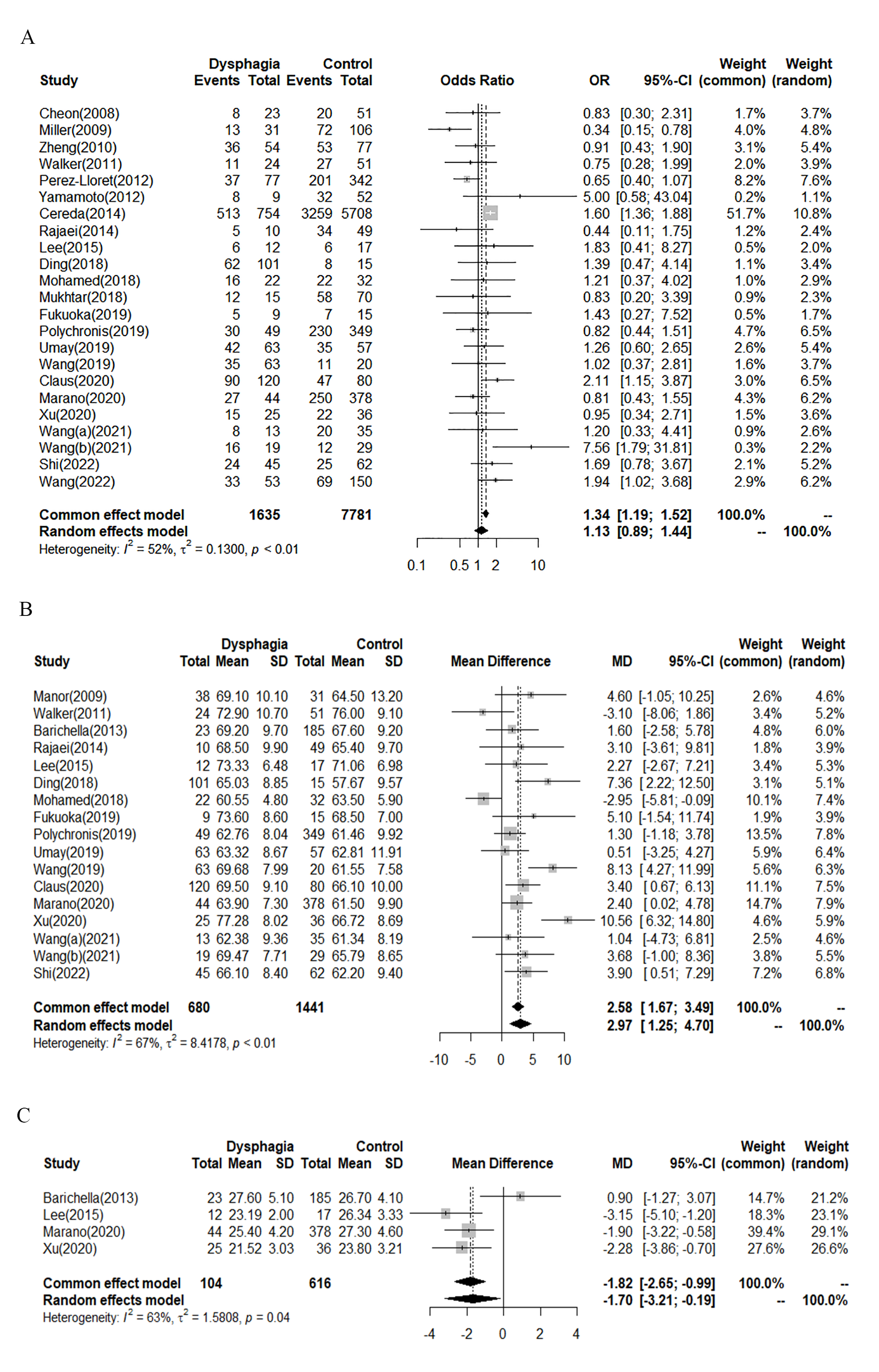

Supplement: Supplementary Figure 4 — The forest plot for gender (A), age (B), BMI (C) based on random-effect analysis. [file Image_4.TIF]

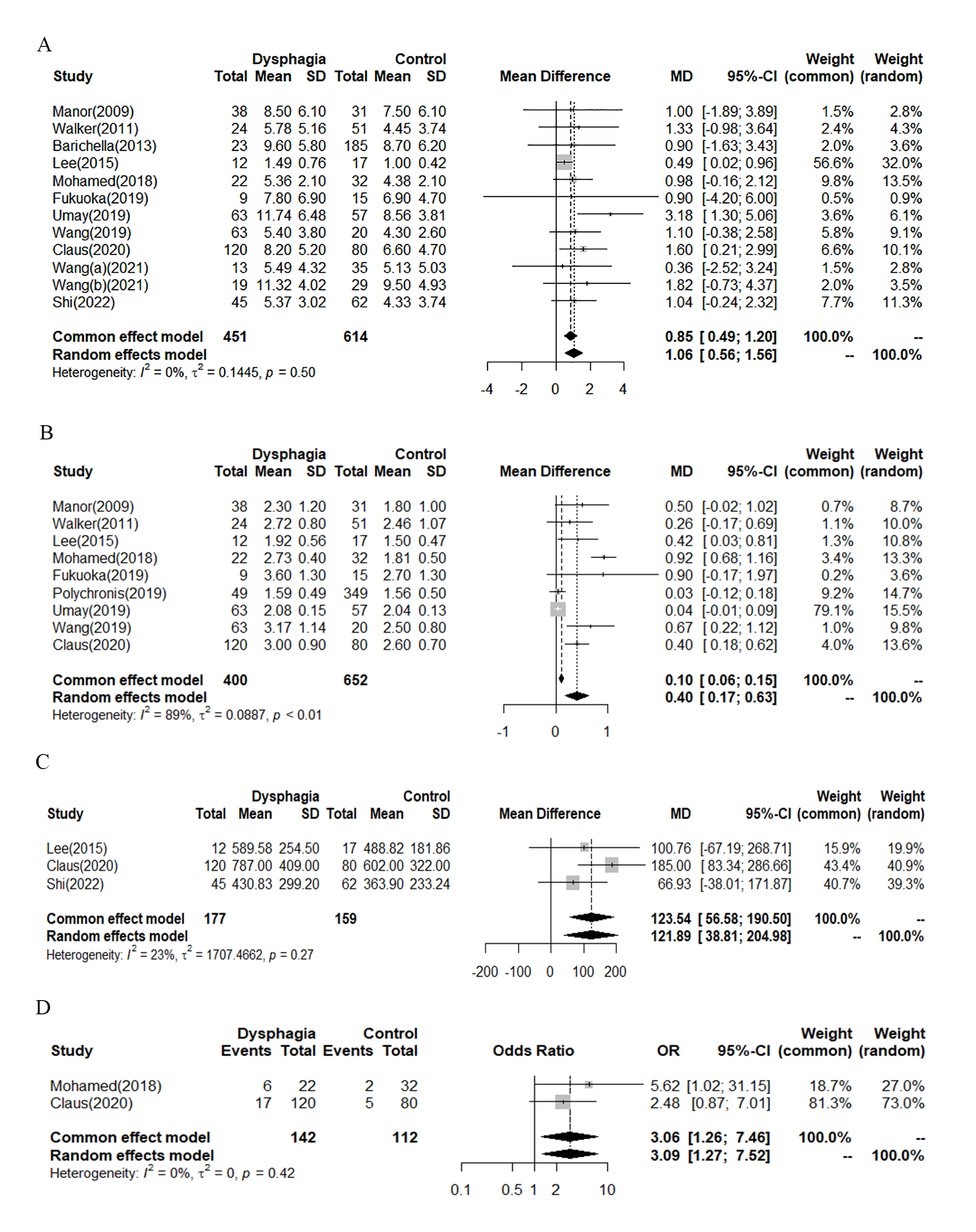

Supplement: Supplementary Figure 5 — The forest plot for disease duration (A), H-Y stage (B), LEDD (C), PIGD subtype (D) based on random-effect analysis. [file Image_5.TIF]

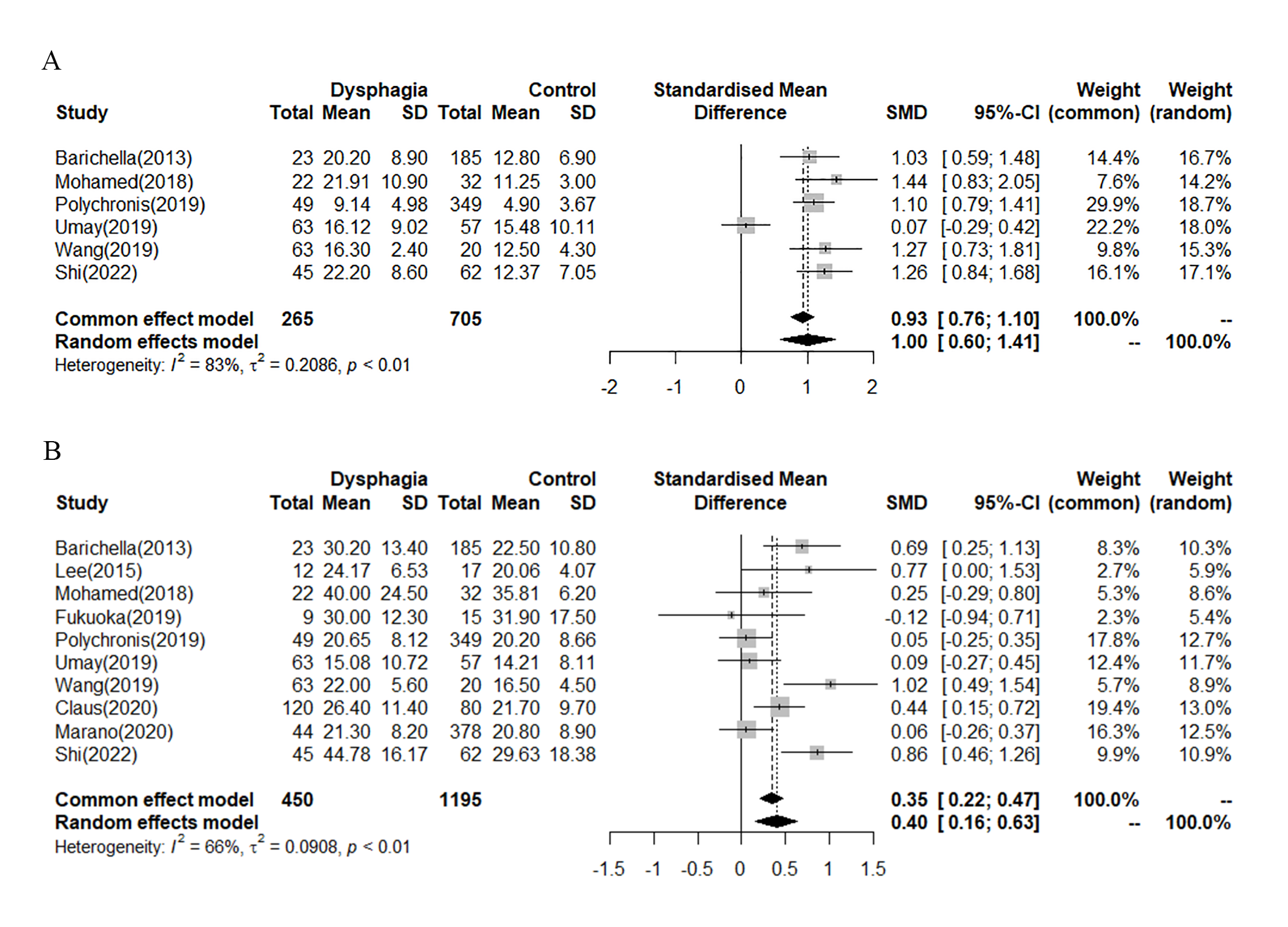

Supplement: Supplementary Figure 6 — The forest plot for UPDRS-II/MDS-UPDRS-II scores (A), UPDRS-III/MDS-UPDRS-III scores (B) based on random-effect analysis. [file Image_6.TIF]

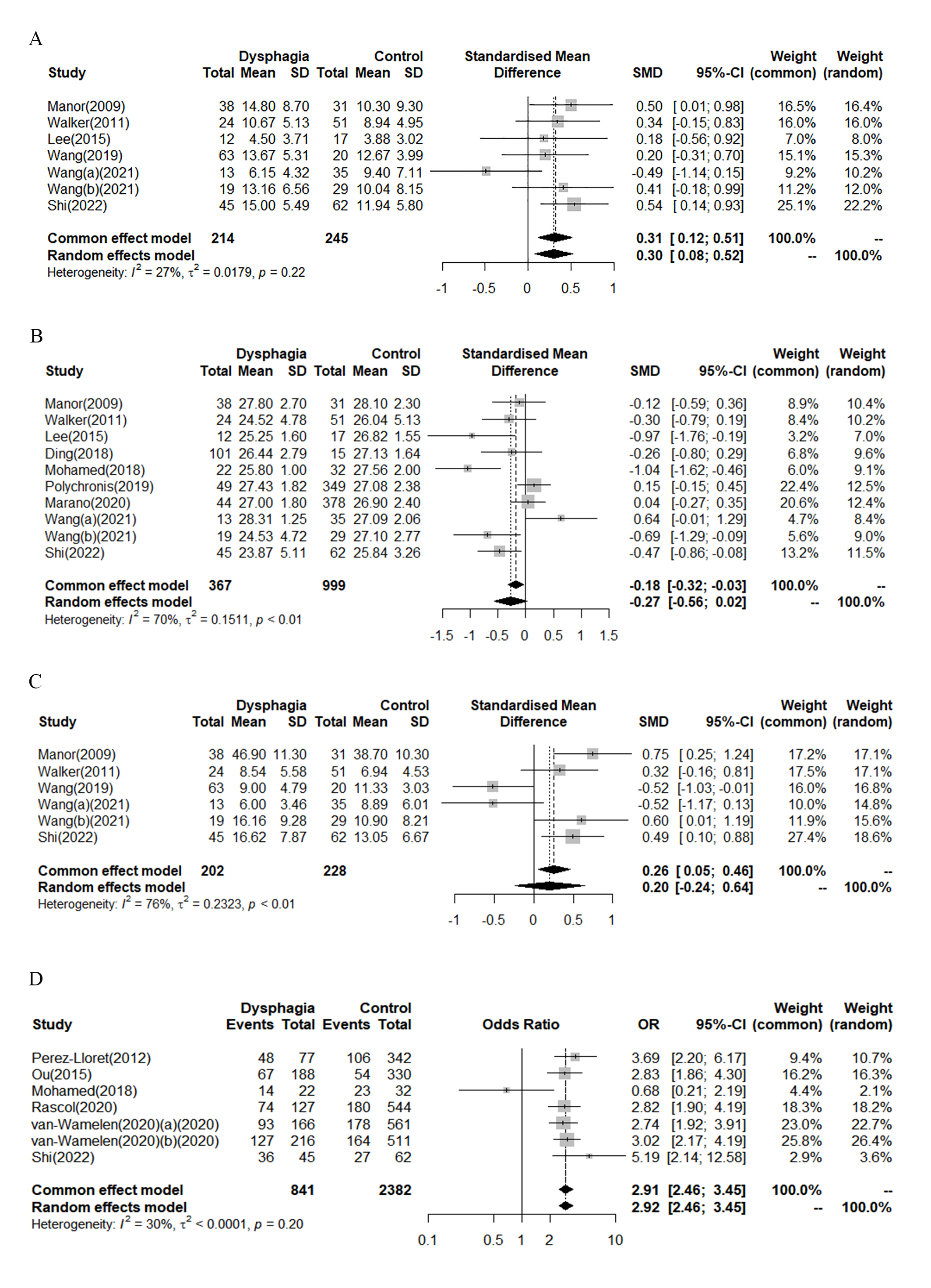

Supplement: Supplementary Figure 7 — The forest plot for depression (A), cognitive function (B), anxiety (C), drooling (D) based on random-effect analysis. [file Image_7.TIF]

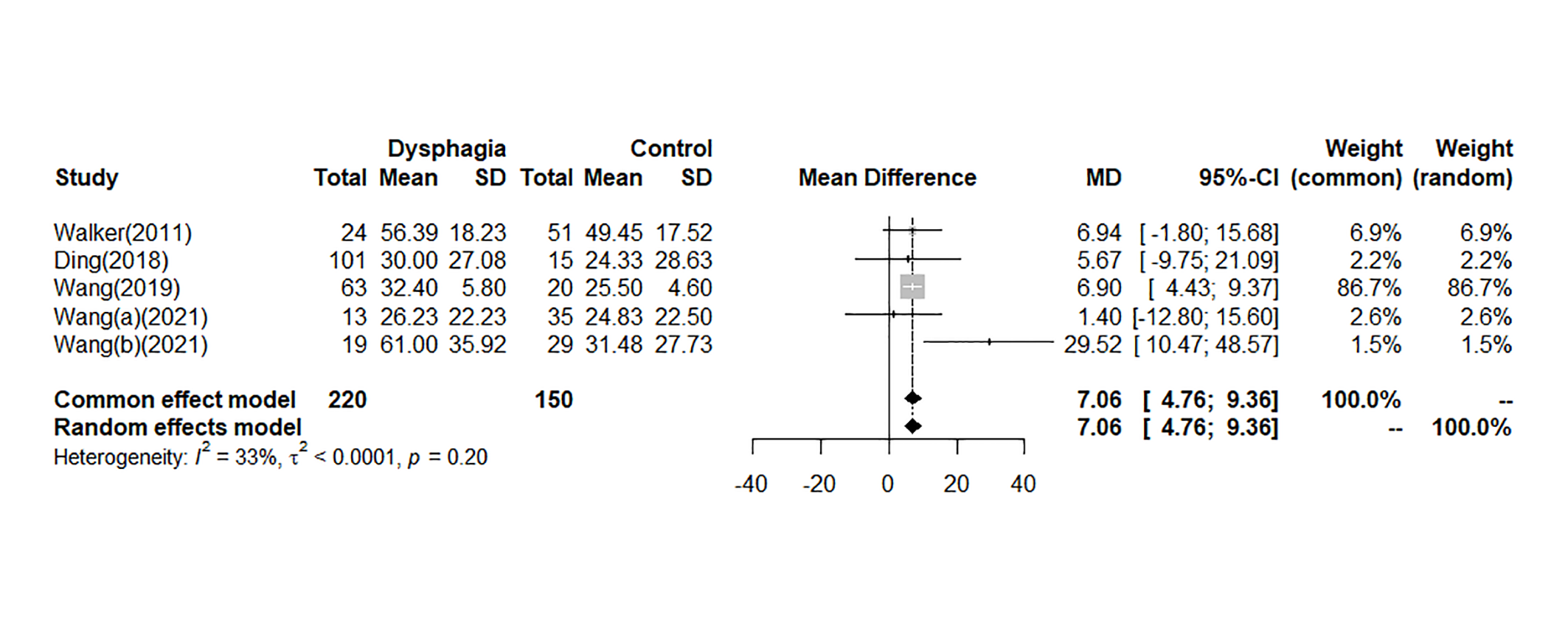

Supplement: Supplementary Figure 8 — The forest plot for PDQ-39 scores based on random-effect analysis. [file Image_8.TIF]
