# Supplementary material for: The prevalence and associated factors of dysphagia in Parkinson's disease: A systematic review and meta-analysis
Source: Front Neurol. 2022 Oct 6;13:1000527. doi: 10.3389/fneur.2022.1000527 (PMC9582284; doi:10.3389/fneur.2022.1000527)
Supplement: Supplementary Table 1 — The detailed search strategy. [file Table_1.DOCX]

**Supplementary Table 1.The detailed search strategy**

| **Search** | **Query** | **Items found** |
| --- | --- | --- |
| **1. Pubmed** | | |
| #1 | “Parkinson Disease”[MesH Terms] | 77,209 |
| #2 | ((“Paralysis Agitans”[Title/Abstract]) OR (PD[Title/Abstract])) OR (Parkinson*[Title/Abstract]) | 252,249 |
| #3 | (#1) OR (#2) | 259,418 |
| #4 | Deglutition Disorders[MeSH Terms] | 57,400 |
| #5 | ((((((dysphagia[Title/Abstract]) OR (“deglutition disorder*”[Title/Abstract])) OR (“swallowing dysfunction”[Title/Abstract])) OR (“swallowing disorder”[Title/Abstract])) OR (“impaired swallowing”[Title/Abstract])) OR (“acataposis”[Title/Abstract])) OR (“swallow problem*”[Title/Abstract]) | 33,994 |
| #6 | (#4) OR (#5) | 74,659 |
| #7 | (#3) AND (#6) | 1,274 |
| **2. Embase** | | |
| #1 | 'paralysis agitans':ti,ab,kw OR pd:ti,ab,kw OR parkinson*:ti,ab,kw | 390,372 |
| #2 | dysphagia:ti,ab,kw OR 'deglutition disorder*':ti,ab,kw OR 'swallowing dysfunction':ti,ab,kw OR 'swallowing disorder':ti,ab,kw OR 'impaired swallowing':ti,ab,kw OR acataposis:ti,ab,kw OR 'swallow problem*':ti,ab,kw | 58,819 |
| #3 | #1 AND #2 | 2,143 |
| **3.Web of Science** | | |
| #1 | ((TS=(Parkinson*)) OR TS=(Paralysis Agitans)) OR TS=(PD) | 615,669 |
| #2 | ((((((TS=(dysphagia)) OR TS=(deglutition disorder*)) OR TS=(swallowing dysfunction)) OR TS=(swallowing disorder)) OR TS=(impaired swallowing)) OR TS=(acataposis)) OR TS=(swallow problem*) | 68,171 |
| #3 | #1 AND #2 | 2,350 |
| **4.the Cochrane Library** | | |
| #1 | MeSH descriptor: [Parkinson Disease] explode all trees | 4,730 |
| #2 | (Parkinson*):ti,ab,kw OR ("Paralysis Agitans"):ti,ab,kw OR (PD):ti,ab,kw | 44,900 |
| #3 | #1 OR #2 | 44,900 |
| #4 | MeSH descriptor: [Deglutition Disorders] explode all trees | 3,114 |
| #5 | (dysphagia):ti,ab,kw OR (deglutition disorder*):ti,ab,kw OR (swallowing dysfunction):ti,ab,kw OR (swallowing disorder):ti,ab,kw OR (impaired swallowing):ti,ab,kw OR (acataposis):ti,ab,kw OR (swallow problem*):ti,ab,kw | 5,384 |
| #6 | #4 OR #5 | 7,360 |
| #7 | #3 AND #6 | 304 |
| **5.CNKI** | | |
| #1 | TKA='帕金森' and TKA=('吞咽障碍'+'吞咽困难'+'咽下困难'+'吞咽功能'+'进食困难'+'进食障碍') | 353 |
| **6.Wanfang data** | | |
| #1 | (全部:(帕金森)) and (全部:(吞咽障碍) or 全部:(吞咽困难) or 全部:(咽下困难) or 全部:(吞咽功能) or 全部:(进食困难) or 全部:(进食障碍)) | 376 |
| **7.SinoMed** | | |
| #1 | "帕金森"[全部字段:智能] AND( "吞咽障碍"[全部字段:智能] OR "吞咽困难"[全部字段:智能] OR "咽下困难"[全部字段:智能] OR "吞咽功能"[全部字段:智能] OR "进食困难"[全部字段:智能] OR "进食障碍"[全部字段:智能]) | 264 |
| **8.VIP** | | |
| #1 | U=(帕金森) AND U=(吞咽障碍 OR 吞咽困难 OR 咽下困难 OR 吞咽功能 OR 进食困难 OR 进食障碍) | 293 |
