# Supplementary material for: The prevalence and associated factors of dysphagia in Parkinson's disease: A systematic review and meta-analysis
Source: Front Neurol. 2022 Oct 6;13:1000527. doi: 10.3389/fneur.2022.1000527 (PMC9582284; doi:10.3389/fneur.2022.1000527)
Supplement: Supplementary Table 2 — Quality assessment based on NOS. [file Table_2.DOCX]

**Supplementary Table 2. Quality assessment based on NOS**

| **Study** | **Study design** | **Selection** | | | | **Comparability** | | **Exposure/**  **Outcome** | | | **NOS** | **Quality** |
| --- | --- | --- | --- | --- | --- | --- | --- | --- | --- | --- | --- | --- |
| Lo (2009) | Cohort | √ | √ | √ |  | √ |  | √ | √ |  | 6 | Moderate |
| Cersosimo (2013) | Case-control | √ | √ | √ |  | √ |  | √ | √ |  | 6 | Moderate |
| Lee (2015) | Cohort | √ | √ | √ | √ | √ |  | √ | √ |  | 7 | High |
| Li (2016) | Cohort | √ | √ | √ |  | √ |  | √ |  |  | 5 | Moderate |
| Fereshtehnejad (2017) | Cohort | √ | √ | √ |  | √ |  | √ | √ |  | 6 | Moderate |
| Malek (2017) | Cohort | √ | √ | √ |  | √ |  | √ |  |  | 5 | Moderate |
| Wang (2017) | Cohort | √ | √ | √ | √ | √ |  | √ |  |  | 6 | Moderate |
| Fukuoka (2019) | Cohort | √ | √ | √ | √ | √ |  | √ |  |  | 6 | Moderate |
| Paul (2019) | Cohort | √ | √ | √ |  | √ |  | √ |  |  | 5 | Moderate |
| Polychronis (2019) | Cohort | √ | √ | √ |  | √ |  | √ |  |  | 5 | Moderate |
| Umay (2019) | Cohort | √ | √ | √ | √ | √ |  | √ |  |  | 6 | Moderate |
| Claus (2020) | Cohort | √ | √ | √ |  | √ |  | √ |  |  | 5 | Moderate |
| Marano (2020) | Cohort | √ | √ | √ |  | √ |  | √ | √ |  | 6 | Moderate |
| Rascol (2020) | Cohort | √ | √ | √ | √ | √ |  | √ | √ |  | 7 | High |
| Schrag (2020) | Cohort | √ | √ | √ |  | √ |  | √ |  |  | 5 | Moderate |
| Wamelen (2020) | Cohort | √ | √ | √ |  | √ |  | √ | √ | √ | 7 | High |
| Cao (2021) | Cohort | √ | √ | √ |  | √ |  | √ |  |  | 5 | Moderate |
| Wang (2021) | Cohort | √ | √ | √ | √ | √ |  | √ | √ | √ | 8 | High |
| Dilmaghani (2022) | Cohort | √ | √ | √ | √ | √ |  | √ |  |  | 6 | Moderate |
| Longardner (2022) | Cohort | √ | √ | √ | √ | √ |  | √ |  |  | 6 | Moderate |
| Vogel (2022) | Cohort | √ | √ | √ | √ | √ |  | √ |  |  | 6 | Moderate |
