# Supplementary material for: The prevalence and associated factors of dysphagia in Parkinson's disease: A systematic review and meta-analysis
Source: Front Neurol. 2022 Oct 6;13:1000527. doi: 10.3389/fneur.2022.1000527 (PMC9582284; doi:10.3389/fneur.2022.1000527)
Supplement: Supplementary Table 3 — Quality assessment based on AHRQ. [file Table_3.DOCX]

**Supplementary Table 3. Quality assessment based on AHRQ**

| **Study** | **Study design** | **1** | **2** | **3** | **4** | **5** | **6** | **7** | **8** | **9** | **10** | **11** | **AHRQ** | **Quality** |
| --- | --- | --- | --- | --- | --- | --- | --- | --- | --- | --- | --- | --- | --- | --- |
| Coates (1997) | Cross-sectional |  |  |  | √ | √ |  |  |  |  |  |  | 2 | Low |
| Clarke (1998) | Cross-sectional | √ |  | √ | √ | √ | √ |  |  |  |  |  | 5 | Moderate |
| Siddiqui (2002) | Cross-sectional |  |  |  | √ | √ |  |  |  |  |  |  | 2 | Low |
| Martin (2007) | Cross-sectional | √ | √ |  | √ | √ | √ |  |  |  |  |  | 5 | Moderate |
| Cheon (2008) | Cross-sectional | √ |  |  | √ | √ | √ |  |  |  |  |  | 4 | Moderate |
| Barone (2009) | Cross-sectional | √ | √ |  | √ | √ | √ |  |  |  |  |  | 5 | Moderate |
| Manor (2009) | Cross-sectional | √ | √ |  | √ | √ | √ |  |  |  |  |  | 5 | Moderate |
| Miller (2009) | Cross-sectional | √ | √ |  | √ | √ | √ |  | √ |  |  |  | 6 | Moderate |
| Zheng (2010) | Cross-sectional | √ | √ | √ | √ | √ | √ |  |  |  |  |  | 6 | Moderate |
| Walker (2011) | Cross-sectional | √ | √ | √ | √ | √ | √ |  |  |  |  |  | 6 | Moderate |
| Lloret (2012) | Cross-sectional | √ | √ |  | √ | √ | √ | √ |  | √ |  |  | 7 | Moderate |
| Yamamoto (2012) | Cross-sectional | √ | √ | √ | √ | √ | √ |  |  |  |  |  | 6 | Moderate |
| Ma (2012) | Cross-sectional | √ | √ | √ | √ | √ |  |  |  |  |  |  | 5 | Moderate |
| Barichella (2013) | Cross-sectional | √ | √ | √ | √ | √ | √ |  | √ |  |  |  | 7 | Moderate |
| Guo (2013) | Cross-sectional | √ | √ | √ | √ | √ | √ |  |  |  |  |  | 6 | Moderate |
| Cereda (2014) | Cross-sectional | √ | √ | √ | √ | √ | √ |  | √ |  |  |  | 7 | Moderate |
| Rajaei (2014) | Cross-sectional | √ | √ | √ | √ | √ | √ |  | √ |  |  |  | 7 | Moderate |
| Simons (2014) | Cross-sectional | √ | √ | √ | √ | √ | √ | √ |  | √ |  |  | 8 | High |
| Ou (2015) | Cross-sectional | √ | √ | √ | √ | √ | √ |  | √ |  |  |  | 7 | Moderate |
| Zhang (2016) | Cross-sectional | √ | √ | √ | √ | √ |  |  |  |  |  |  | 5 | Moderate |
| Barbe (2017) | Cross-sectional | √ | √ | √ | √ | √ |  |  |  |  |  |  | 5 | Moderate |
| Ding (2018) | Cross-sectional | √ | √ | √ | √ | √ | √ |  |  |  |  |  | 6 | Moderate |
| Mohamed (2018) | Cross-sectional | √ | √ | √ | √ | √ | √ |  | √ |  |  |  | 7 | Moderate |
| Mukhtar (2018) | Cross-sectional | √ | √ | √ | √ | √ |  |  |  |  |  |  | 5 | Moderate |
| Barbe (2019) | Cross-sectional | √ | √ | √ | √ | √ |  |  |  |  |  |  | 5 | Moderate |
| Nienstedt (2019) | Cross-sectional | √ | √ | √ | √ | √ | √ |  | √ |  |  |  | 7 | Moderate |
| Oad (2019) | Cross-sectional | √ | √ | √ | √ | √ | √ |  |  |  |  |  | 6 | Moderate |
| Wang (2019) | Cross-sectional | √ | √ | √ | √ | √ | √ |  |  |  |  |  | 6 | Moderate |
| Bakhtiyari (2020) | Cross-sectional | √ | √ | √ | √ | √ | √ |  |  |  |  |  | 6 | Moderate |
| Fagerberg (2020) | Cross-sectional | √ | √ | √ | √ | √ | √ |  |  |  |  |  | 6 | Moderate |
| Lin (2020) | Cross-sectional | √ | √ | √ | √ | √ |  |  |  |  |  |  | 5 | Moderate |
| Wang (2020) | Cross-sectional | √ | √ | √ | √ | √ |  |  |  |  |  |  | 5 | Moderate |
| Xu (2020) | Cross-sectional | √ | √ | √ | √ | √ |  |  |  |  |  |  | 5 | Moderate |
| Ayele (2021) | Cross-sectional | √ | √ | √ | √ | √ | √ |  |  |  |  |  | 6 | Moderate |
| Frank (2021) | Cross-sectional | √ | √ | √ | √ | √ | √ | √ | √ |  |  |  | 8 | High |
| Shi (2022) | Cross-sectional | √ | √ | √ | √ | √ | √ |  | √ |  |  |  | 7 | Moderate |
| Wang (2022) | Cross-sectional | √ | √ | √ | √ | √ |  |  |  |  |  |  | 5 | Moderate |
